# Obituary

**Published:** 1894-10

**Authors:** 


### Obituary.

Dr. Albert B. Miles, of New Orleans, professor of therapeutics, materia medica and hygiene in the medical department of the University of Louisiana, died after a brief illness August 5, 1894. Dr. Miles was chairman of the committee of arrangements of the Southern Surgical and Gynecological Association at its New Orleans meeting, November, 1893, and through his active labors contributed greatly to the success of the meeting. He was elected vice-president of the association, which office he held at the time of his death. Dr. Miles will be greatly missed, not only in New Orleans, but throughout the South, where he was widely and well known. He is reported to have left an estate valued at $250,000, amassed during a practice of twenty years. Under his will the charity hospital, the Hotel Dieu and the medical school of Tulane University of New Orleans will receive $10,000.

———

Dr. William Cecil Dabney, professor of obstetrics and of practice of medicine at the University of Virginia, died at Charlottesville, August 20, 1894, of typhoid fever, aged 45 years. Dr. Dabney was eminent in his profession and had received many honors at its hands. He was a prominent member of the Southern Surgical and Gynecological Association.

